# Zinc finger and SCAN domain containing 1, ZSCAN1, is a novel stemness-related tumor suppressor and transcriptional repressor in breast cancer targeting TAZ

**DOI:** 10.3389/fonc.2023.1041688

**Published:** 2023-02-27

**Authors:** Jian Chu, Yunzhe Li, Misi He, Hui Zhang, Lingling Yang, Muyao Yang, Jingshu Liu, Chenxi Cui, Liquan Hong, Xingchi Hu, Lei Zhou, Tangya Li, Changchun Li, Huiwen Fan, Guoqin Jiang, Tingyuan Lang

**Affiliations:** ^1^ Department of Surgery, The Second Affiliated Hospital of Soochow University, Suzhou, Jiangsu, China; ^2^ College of Bioengineering, Chongqing University, Chongqing, China; ^3^ Department of Gynecologic Oncology, Chongqing University Cancer Hospital & Chongqing Cancer Institute & Chongqing Cancer Hospital, Chongqing, China; ^4^ Department of Breast Cancer Center, Chongqing University Cancer Hospital, Chongqing, China; ^5^ School of Medicine, Chongqing University, Chongqing, China; ^6^ Obstetrics and Gynecology Department, The Second Affiliated Hospital of Chongqing Medical University, Chongqing, China; ^7^ Department of Clinical Laboratory, Affiliated Hospital of Hangzhou Normal University, Hangzhou, Zhejiang, China; ^8^ Department of General Surgery, Yancheng City No.1 People’s Hospital, Yancheng, Jiangsu, China; ^9^ School of Optometry, Department of Applied Biology and Chemical Technology, Research Centre for SHARP Vision (RCSV), The Hong Kong Polytechnic University, Hong Kong, Hong Kong SAR, China; ^10^ Centre for Eye and Vision Research (CEVR), 17W Hong Kong Science Park, Hong Kong, Hong Kong SAR, China; ^11^ Reproductive Medicine Center, The First Affiliated Hospital of Chongqing Medical University, Chongqing, China

**Keywords:** ZSCAN1, breast cancer, stemness, tumor suppressor, transcriptional repressor, TAZ

## Abstract

**Introduction:**

Cancer stem cells (CSCs) targeted therapy holds the potential for improving cancer management; identification of stemness-related genes in CSCs is necessary for its development.

**Methods:**

The Cancer Genome Atlas (TCGA) and the Molecular Taxonomy of Breast Cancer International Consortium (METABRIC) datasets were used for survival analysis. ZSCAN1 correlated genes was identified by Spearman correlation analysis. Breast cancer stem-like cells (BCSLCs) were isolated by sorting CD44+CD24- cells from suspension cultured breast cancer (BC) spheroids. The sphere-forming capacity and sphere- and tumor-initiating capacities were determined by sphere formation and limiting dilution assays. The relative gene expression was determined by qRT-PCR, western blot. Lentivirus system was used for gene manipulation. Nuclear run-on assay was employed to examine the levels of nascent mRNAs. DNA pull-down and Chromatin immunoprecipitation (ChIP) assays were used for determining the interaction between protein and target DNA fragments. Luciferase reporter assay was used for evaluating the activity of the promoter.

**Results and discussion:**

ZSCAN1 is aberrantly suppressed in BC, and this suppression indicates a bad prognosis. Ectopic expression of ZSCAN1 inhibited the proliferation, clonogenicity, and tumorigenicity of BC cells. ZSCAN1-overexpressing BCSLCs exhibited weakened stemness properties. Normal human mammary epithelial (HMLE) cells with ZSCAN1 depletion exhibited enhanced stemness properties. Mechanistic studies showed that ZSCAN1 directly binds to -951 ~ -925bp region of WWTR1 (encodes TAZ) promoter, inhibits WWTR1 transcription, thereby inhibiting the stemness of BCSCs. Our work thus revealed ZSCAN1 as a novel stemness-related tumor suppressor and transcriptional repressor in BC.

## Introduction

Breast cancer (BC) is still the most common cancer type and a lethal primary malignant tumor worldwide (1). BC is a heterogeneous disease at both molecular and clinical level (2). Usually, BC was divided into luminal, HER2-amplified, and triple-negative/basal cancers (3, 4). Luminal BC, the most common subtype, has the best prognosis and benefits from targeted endocrine therapies due to hormone receptor expression. HER2-amplified BC benefits from anti-HER2 therapies. While triple-negative BC has the poorest prognosis and is commonly treated with traditional cytotoxic chemotherapeutic drugs in the absence of a therapeutic target.

Recently, an evolutionary concept in heterogeneous BC has been well discussed that BC tissues are hierarchically organized, in which a small subpopulation of BC stem cells (BCSCs) contributes to growth and progression, in contrast, most cancer cells in the tumor are non- or poor-tumorigenic which are derived from BCSCs through mechanical changes, such as epigenetic regulation (5, 6). Consistent with the cancer stem cell (CSC) model, an effective therapeutic strategy for BC should focus on BCSCs instead of traditional therapies that attempt to eliminate all BC cells (7). To achieve this goal, several fundamental signaling pathways involved in BC stemness maintenance, such as Hippo, Wnt, and hedgehog (Hh), have been revealed (8, 9). However, although massive studies, our understandings of the regulation of BC stemness are still incompletely understood.

Zinc finger proteins (ZNFs) constitute a large class of transcription factors regulating the plasticity of human stem cells, including CSCs (10–12). The C_2_H_2_ finger domain is one of the classical zinc finger domains which defines a subgroup of ZNFs named C_2_H_2_ family Zinc finger proteins (13, 14). While, SCAN domain, which mainly exists in C_2_H_2_ family zinc finger proteins, defines another subgroup named SCAN domain containing Zinc finger or Zinc finger and SCAN domain containing (ZSCAN) transcription regulators (15). The SCAN domain, also known as the leucine rich region (LeR), is a conserved 84 residue motif; more than 71 domains have been identified in the human genome (15). However, although several studies have reported the association of ZSCAN family members with lipid metabolism, cell growth, and differentiation (16–18), the function of most members in this group has remained elusive.

Evidence has recently shown abnormal methylation and expression of ZSCAN1 in cancer tissues (19–21), but the critical role of this dysregulation is unknown. As a result, the goal of this study is to investigate the functional role of ZSCAN1 in BC.

## Materials and methods

### Analysis with public datasets

Both automatic and manual survival analyses with The Cancer Genome Atlas (TCGA) dataset were performed. Automatic survival analysis was performed by Xena online platform (22); GDC TCGA Breast Cancer (BRCA) dataset was chosen, in which gene expression data (RNA seq) from 1217 samples from TCGA program are included, and both median and quartiles of ZSCAN1 expression were set up as cut points. Manual survival analysis was performed according to the workflow previously described (23). Briefly, the RNA sequencing data was downloaded, and the missing values were first imputed by KNN (N=5) program. Normalization was performed by the Quantile normalization algorithm. The missing values were then imputed again by KNN (N=5) program, followed by scaling using Z-score scaling program. Kaplan-Meier analysis was used for survival analysis, and similarly, both median and quartiles of ZSCAN1 expression were set up as cut points. The data from the Molecular Taxonomy of Breast Cancer International Consortium (METABRIC) (24) was obtained from the cBioportal online platform (25); Breast Cancer (METABRIC, Nature 2012 & Nat Commun 2016) (24, 26) dataset, containing targeted sequencing of 2509 primary breast tumors with 548 matched normal tissues, was downloaded for Kaplan-Meier survival analysis. Pearson correlation analysis was employed for screening ZSCAN1 correlated genes with the TCGA dataset; genes with R > 0.15 or R < -0.15 and Ƥ < 0.05 were recognized as correlated genes. Patients clustering was performed by the k-means clustering program (23). Pathways impacted by ZSCAN1 correlated genes were identified by Reactome online tool, which were subsequentially ranked based on false discovery rate (FDR) (27).

### BC samples

In total, 92 BC and 30 normal local tissues were collected from the Department of Surgery in the Second Affiliated Hospital of Soochow University (Suzhou, Jiangsu, China). All procedures were performed in accordance with the Institutional Review Board of the Second Affiliated Hospital of Soochow University (Suzhou, Jiangsu, China). Informed written consent was obtained from each patient.

### Immunohistochemistry and immunohistochemical scoring

The immunohistochemistry was performed according to the standard protocol (28). The immunoreactive score (IRS) scoring method was employed to determine the expression level of the proteins tested as described by Specht, E and colleagues (28).

### Cell culture, primers, antibodies, siRNAs, gRNAs, plasmids, and reagents

Human Mammary Epithelial Cells (HMLE) and human breast cancer cells (MCF-7, T47D, BT474, MDA231, DMA468) were purchased from American Type Culture Collection (ATCC, Manassas, Virginia, USA). HMLEs were cultured in HuMEC Basal Serum-Free Medium (12753018 Thermo Fisher, Waltham, MA, USA) supplemented with the reagents provided in Mammary Epithelial Cell Growth Kit (PCS-600-040 ATCC, Manassas, Virginia, USA) (5 µg mL^-1^ rh-insulin, 6 mM L-Glutamine, 1 µg mL^-1^ Epinephrine, 5 µg mL^-1^ Apo-Transferrin, 5 ng mL^-1^ r-H-TGF-α, 0.4% ExtractP, 100 ng mL^-1^ Hydrocortisone Hemmisuccinate). MCF-7 cells were cultured in Eagle’s Minimum Essential Medium supplemented with 0.01 mg ml^-1^ human recombinant insulin and 10% fetal bovine serum. T47D cells were cultured in RPMI-1640 medium supplemented with 0.1 units ml^-1^ bovine insulin and 10% fetal bovine serum. BT474 cells were cultured in Hybri-Care medium supplemented with 1.5 g L^-1^ sodium bicarbonate and 10% fetal bovine serum. MDA231 and MDA468 cells were cultured in Leibovitz’s medium supplemented with 10% fetal bovine serum. The mycoplasma status of the cells was routinely checked and was confirmed to be negative. For spheroid culture, cell indicated cells were suspension cultured in ultra-low attachment culture dish/plate (3471/4520, Corning, Inc, Corning, NY, USA) containing serum-free medium supplemented recombinant human epidermal growth factor (8 ng ml^-1^), recombinant human basic fibroblast growth factor (8 ng ml^-1^), glucocorticoid (0.2 μg ml^-1^) and B27 (1.5%). All the medium and reagents involved in cell culture were purchased from Thermo Fisher except where indicated. Lentivirus plasmids pCDH-CMV-MCS-EF1-Puro was kindly provided by Professor Hongbin Ji (Shanghai Institutes for Biological Sciences, Chinese Academy of Sciences, Shanghai, China). gRNAs were inserted into lentiGuide-Puro (#52963 Addgene, Cambridge, MA, USA). siRNAs (AM16708 for ZSCAN1, SIC001 for control) were purchased from Sigma-Aldrich (St. Louis, MO, USA). 8xGTIIC-*luciferase plasmid (#34615 A*ddgene, Cambridge, MA, USA*) was used for determining TAZ transcriptional activity.* pGL4.20 (E6751 Promega, Madison, WI, USA) was used for routine luciferase reporter assay. Antibodies, primers, and gRNAs were listed in **Supplementary Tables 1–3**.

### Quantitative reverse transcription polymerase chain reaction

The qRT-PCR assay was performed according to the standard protocol as previously reported (29). The RNA samples were prepared by isolation with Trizol reagent (10296010 Thermo Fisher, Waltham, MA, USA). Ethanol was removed by evaporation. The residual DNA was removed by Turbo DNAse (AM2239 Thermo Fisher, Waltham, MA, USA). SuperScript™ III Platinum™ (11732088 Thermo Fisher, Waltham, MA, USA) was used to perform qRT-PCR assay. *GAPDH* was used as a normalization control.

### CD44^+^CD24^-^ cells sorting

Magnetic Assisted Cell Sorting (MACS) technology (Miltenyi Biotech, Bergisch Gladbach, Germany) was used for the isolation of CD44^+^CD24^-^ cells. Briefly, the suspension cultured spheroids were dissociated first. The cells were then labelled with CD44/CD24-Biotin antibody and incubated at 4°C for 15 mins. The labelled cells were washed and further labelled with anti-biotin microbeads. Magnetic separation was carried out by using LS columns arranged in the magnetic field of a magnetic separator. CD44/CD24 negative cells were collected in flowthrough, oppositely, CD44/CD24 positive enriched fraction was obtained by flushing out labelled cells from the column. The labels were removed by the microbead Release reagent. The MACS sorted cell populations were tested using flow cytometry.

### Xenografts

Five to six-week-old BALB/c nude female mice were subcutaneously injected with a 100 μl of 5 × 10^6^ cells suspended in the 1:1 mixture of serum-free medium and matrigel (356234, Corning, Inc, Corning, NY, USA) in the flank. The size of the xenograft was measured at least once a week. Mice were sacrificed when the termination criteria were reached. All the procedures were approved by Institutional Animal Care and Use Committee in The Second Affiliated Hospital of Soochow University (Suzhou, Jiangsu, China).

### Limiting dilution assay

*In vitro* and *in vivo* limiting dilution assay is commonly used to determine the cancer initiating cell frequency of the tested cancer cells (30, 31). Briefly, for *in vitro* assay, the cells were plated at one, five, and 10 cells per well into an ultra-low attachment plate (50 wells for each group). The next day, each well was visually checked for the presence of an indicated number of cells. Fifteen days after plating, the number of wells containing spheroids were quantified by manual counting. On the other hand, for *in vivo* assay, eight, 40, and 200 cells were injected subcutaneously into the mice model (10 mice for each group). The tumors were monitored every week, and the number of mice bearing tumor was quantified at the endpoint. Extreme limiting dilution assay analyses (ELDAs) were performed by ELDA online software designed by Hu and colleagues (32).

### Sphere formation assay

Cells were placed in a spheroid culture medium (mentioned above). Suspended cells were seeded into ultra-low-attachment 6-well plates (Corning, Inc., Corning, NY, USA) at a density of 3,000 cells/well and incubated at 37 °C in a 5% CO2-humidified incubator. After 15 days, spheres were quantitated using inverted phase-contrast microscopy and photographed.

### Cell viability test

Cell viability was determined by CellTiter-Glo 3D cell viability assay (Promega) and Trypan blue exclusion assay.

### Cytoplasmic and nuclear protein extraction

Cytoplasmic and nuclear protein extraction was performed by NE-PER Nuclear and Cytoplasmic Extraction Reagents (78833, Thermo Fisher, Waltham, MA, USA), which contains 10 mL Cytoplasmic Extraction Reagent I (CERI), 550 μL Cytoplasmic Extraction Reagent II (CER II), and 5 mL Nuclear Extraction Reagent (NER), according to the manual.

### Western blotting

The western blot assay was performed according to the standard protocol previously reported (29). Briefly, the protein samples were first separated by standard sodium dodecyl sulfatepolyacrylamide (SDS) gel electrophoresis, followed by polyvinylidene fluoride (PVDF) membrane (88518 Thermo Fisher, Waltham, MA, USA) transfer. After blocking with blocking buffer, the membranes were incubated with primary antibodies at 4 °C overnight, followed by hybridization with secondary antibodies at room temperature for 2 h. Enhanced chemiluminescence (32134 Thermo Fisher, Waltham, MA, USA) was used to visualize the signal. The antibodies used in this study were listed in supplementary materials.

### Nuclear run-on assay

Click-iT™ Nascent RNA Capture Kit (C10365 Thermo Fisher, Waltham, MA, USA) was used to determine the transcription. Briefly, the nascent RNA was labeled by incubating the cells with 5-ethynyl uridine (EU). The labeled nascent RNA was then separated by biotinylation with biotin azide coupled with affinity-based separation with streptavidin-coupled magnetic beads. The abundance of nascent RNA was determined by qRT-PCR.

### DNA pull-down assay

The promoter of WWTR1 was amplified by PCR and labeled by biotin with Biotin 3’ End DNA labeling Kit (89818, Thermo Fisher Scientific) for immobilization on DynabeadsTM M-270 Streptavidin (65305, Thermo Fisher Scientific). The beads coated with promoters were then incubated with protein extraction from the target cells. The levels of ZSCAN1 in proteins separated by affinity with WWTR1 promoter were determined by western blot.

### Chromatin immunoprecipitation assay

MAGnify™ Chromatin IP System (492024 Thermo Fisher, Waltham, MA, USA) was used for ChIP assay. Briefly, the cells were first crosslinked with formaldehyde, followed by lysis, sonication, and centrifugation for the harvest of protein-crosslinked chromatin. The antibody-coupled beads were subsequentially used for the separation of DNA fragments crosslinked with antibody-specific proteins. The levels of target DNAs were determined by qRT-PCR.

### Luciferase reporter assay

The plasmids containing the luciferase gene driven by promoters were transfected into cells together with pRL-TK plasmids. Dual-luciferase reporter assay system (e1901 Promega Corporation, Madison, WI, USA) was employed to determine the luciferase activity.

### Statistics

The data were presented as mean ± SD. Student’s t-test and one-way ANOVA were used for the determination of the statistical significance between two and multiple groups. P < 0.05 was considered as statistical significance.

## Results

### ZSCAN1 is a novel tumor suppressor in BC

**Figure 1:**
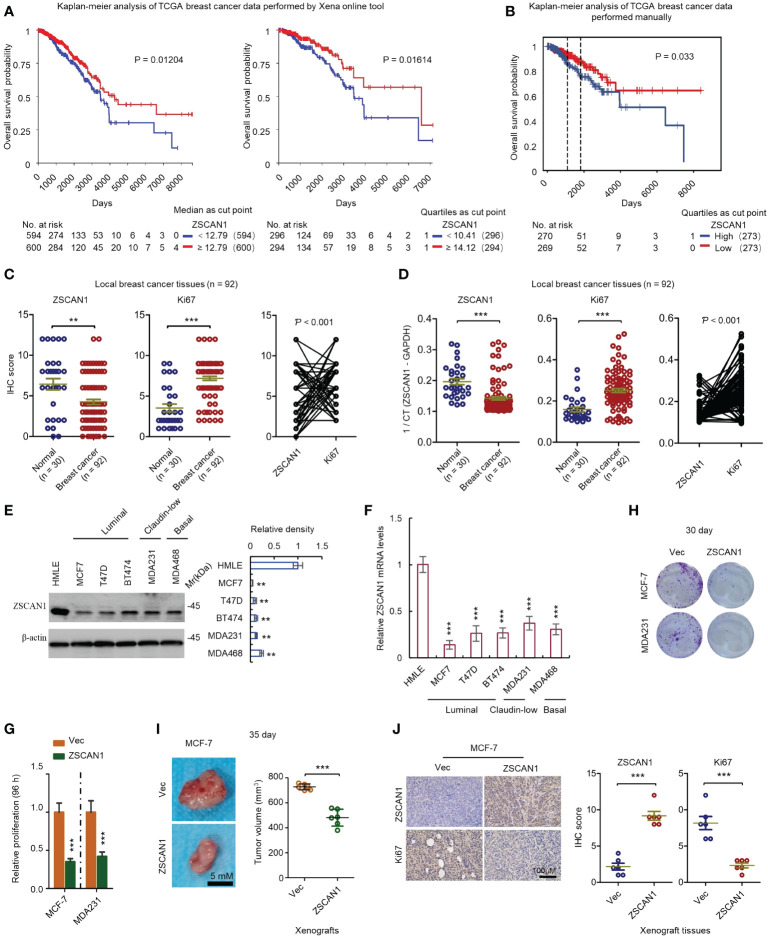
ZSCAN1 plays a tumor suppressor role in breast cancer. **(A, B)** The correlation between ZSCAN1 expression level and breast cancer survival was analyzed by Kaplan-meier analysis with TCGA data by Xena platform **(A)** and manual analysis **(B)**. **(C)** The protein levels of ZSCAN1 and Ki67 in tumor and normal tissues of local patients were analyzed by immunohistochemistry (student’s *t* test, n = 92 for tumor tissues and 30 for normal tissues). The correlation between ZSCAN1 and Ki67 was analyzed by Pearson analysis. **(D)** The mRNA levels of ZSCAN1 and Ki67 in tumor and normal tissues of local patients were analyzed by qRT-PCR (student’s *t* test, n = 92 for tumor tissues and 30 for normal tissues). The correlation between ZSCAN1 and Ki67 was analyzed by Pearson analysis. **(E)** Western blot analysis of the protein levels of ZSCAN1 in normal and breast cancer cell lines (one-way ANOVA, n = 3). **(F)** qRT-PCR analysis of the mRNA levels of ZSCAN1 in normal and breast cancer cell lines (one-way ANOVA, n = 3). **(G)** ZSCAN1 inhibited the proliferation of breast cancer cells as identified by cell viability assay. **(H)** ZSCAN1 inhibited the clonogenicity of breast cancer cells as identified by clonogenic assay (student’s *t* test, n = 3). **(I)** ZSCAN1 inhibited the tumorigenicity of breast cancer cells as identified by xenograft model (student’s *t* test, n = 6). **(J)** Immunohistochemistry analysis revealed that ZSCAN1 inhibited the expression of ki67 in tumor tissues of xenograft model (student’s *t* test, n = 6). **P < 0.01, ***P < 0.001.

To investigate the clinical relevance of ZSCAN1 in BC, we performed a Kaplan-Meier analysis by using the TCGA database (BC RNA-seq data). Xena platform online tool (22) was first used for this analysis. The results showed that no matter whether median or quartiles were used for grouping, patients with higher ZSCNA1 expression exhibits significantly higher overall survival rate versus patients with low ZSCAN1 expression (**Figure 1A**, *P* = 0.01204 for median; *P* = 0.01614 for quartiles). We next downloaded the BC expression data (RNA-seq) from TCGA and manually preprocessed the data as previously described (23), followed by Kaplan-Meier analysis. Consistent with the analysis from the Xena platform, the overall survival rate was significantly increased in patients with high ZSCNA1 expression versus patients with low ZSCAN1 expression (*P* = 0.033) when patients were grouped by the quartiles of ZSCNA1 mRNA expression level (**Figure 1B**). Despite the statistical significance of difference was not observed when grouping patients with the median, a trend toward a better survival was observed in patients with high ZSCAN1 expression (**Supplementary Figure 1A**); the difference between the two analysis is likely due to the different preprocessing strategies adopted (23). We next analyzed ZSCAN1 expression in BC and normal tissues with 92 BC and 30 normal local tissues. Both immunohistochemistry and qRT-PCR results indicated the lower ZSCAN1 expression in BC tissues versus normal tissues (*P* = 0.0063 for immunohistochemistry; *P* < 0.001 for qRT-PCR) (**Figures 1C, D**). Consistently, we next measured the expression of ZSCAN1 in BC and normal cell lines and both western blot and qRT-PCR showed the decreased ZSCAN1 expression in BC cell lines, including basal (MDA468), luminal (MCF7, T47D, and BT474), and claudin-low (MDA231) cells (33), versus HMLE normal cell line (34) (**Figure 1E**, *P* < 0.01 for western in all cells; **Figure 1F**, *P* < 0.001 for qRT-PCR in all cells), indicating the tumor suppressor role of ZSCAN1 in BC.

We next determined whether ZSCAN1 regulates the proliferation of BC cells. MCF-7 and MDA231 were used as a model and ZSCAN1-overexpressing cells were established by a lentivirus delivery system (**Supplementary Figure 1B**). We found that the proliferation was significantly decreased in ZSCAN1-overexpressing MCF-7 and MDA231 cells versus empty vector control cells (**Figure 1G**, n = 3, *P* < 0.001 in MCF-7 and MDA231), as indicated by cell viability assay. Additionally, the clonogenicity was also significantly decreased in the cells with ZSCAN1 overexpression versus control cells (**Figure 1H**; **Supplementary Figure 1C**, n = 3, *P* < 0.001 in MCF-7 and MDA231). Moreover, the volume of the xenografts derived from ZSCAN1-overexpressing MCF-7 cells was significantly smaller than that derived from control cells (**Figure 1I**, n = 6, *P* < 0.001). Furthermore, by both immunohistochemistry and qRT-PCR analysis with local samples, the expression of ZSCAN1 was found to be negatively correlated with Ki67 (**Figure 1C** right, *P* < 0.001, and **Figure 1D** right, *P* < 0.001); Ki67 was found to be elevated in BC tissues versus normal tissues (**Figure 1C** middle, *P* < 0.001, and **Figure 1D** middle, *P* < 0.001). Consistently, the result from immunohistochemistry indicated that the expression of Ki67 was inhibited in xenografts derived from ZSCAN1-overexpressing MCF-7 cells (**Figure 1J**, n = 6, *P* < 0.001). The elevation of ZSCAN1 expression in ZSCAN1-overexpressing xenografts was confirmed by immunohistochemistry (**Figure 1J**, n = 6, *P* < 0.001). Taken together, these results support that ZSCAN1 is suppressed in BC cancer cells, associated with good prognosis, and ectopic expression of ZSCAN1 inhibits the proliferation of BC cells.

### ZSCAN1 inhibits the stemness of BCSLCs

We next investigated the expression of ZSCAN1 in subtypes of BC tissues with the TCGA dataset. We noticed that although a trend toward better prognosis in patients bearing BC with high ZSCAN1 expression was found in all subtypes, the statistical significances of these differences were not observed (**Supplementary Figure 1D**). Similar results were obtained by the same analysis with the dataset from METABRIC database (**Supplementary Figure 2A**) (24–26). Furthermore, when analysis with paired normal and BC tissues in the TCGA dataset, significant downregulation in ZSCAN1 expression was only observed in the basal subtype (**Supplementary Figure 2B**). These results indicate that the effects of ZSCAN1 are not well reflected by the data derived from bulk sequencing (35). We thus speculated that ZSCAN1 may function mainly in a small cell subpopulation in BC tissues, such as BCSCs (36). Thus, to investigate the role of ZSCAN1 in BCSCs, we first isolated BCSLCs from basal, luminal, and claudin-low cells by suspension culture followed by CD44 and CD24 sorting (37) (**Supplementary Figure 3A**). All BCSLCs exhibited increased sphere-forming capacity (**Supplementary Figure 3B**, *P* < 0.001 in all cells) and higher CD44 (**Supplementary Figure 3C**, *P* < 0.001 in all cells) and lower CD24 expression (**Supplementary Figure 3D**, *P* < 0.001 in all cells) versus their adherent cultured parental cells, while, no significant difference in cell viability was observed in the spheres (**Supplementary Figure 3E**), indicating that isolated BCSLCs exhibit significantly higher stemness properties versus adherent parental cells, and therefore, the isolated BCSLCs could be served as a cell model for BCSCs study.

**Figure 2:**
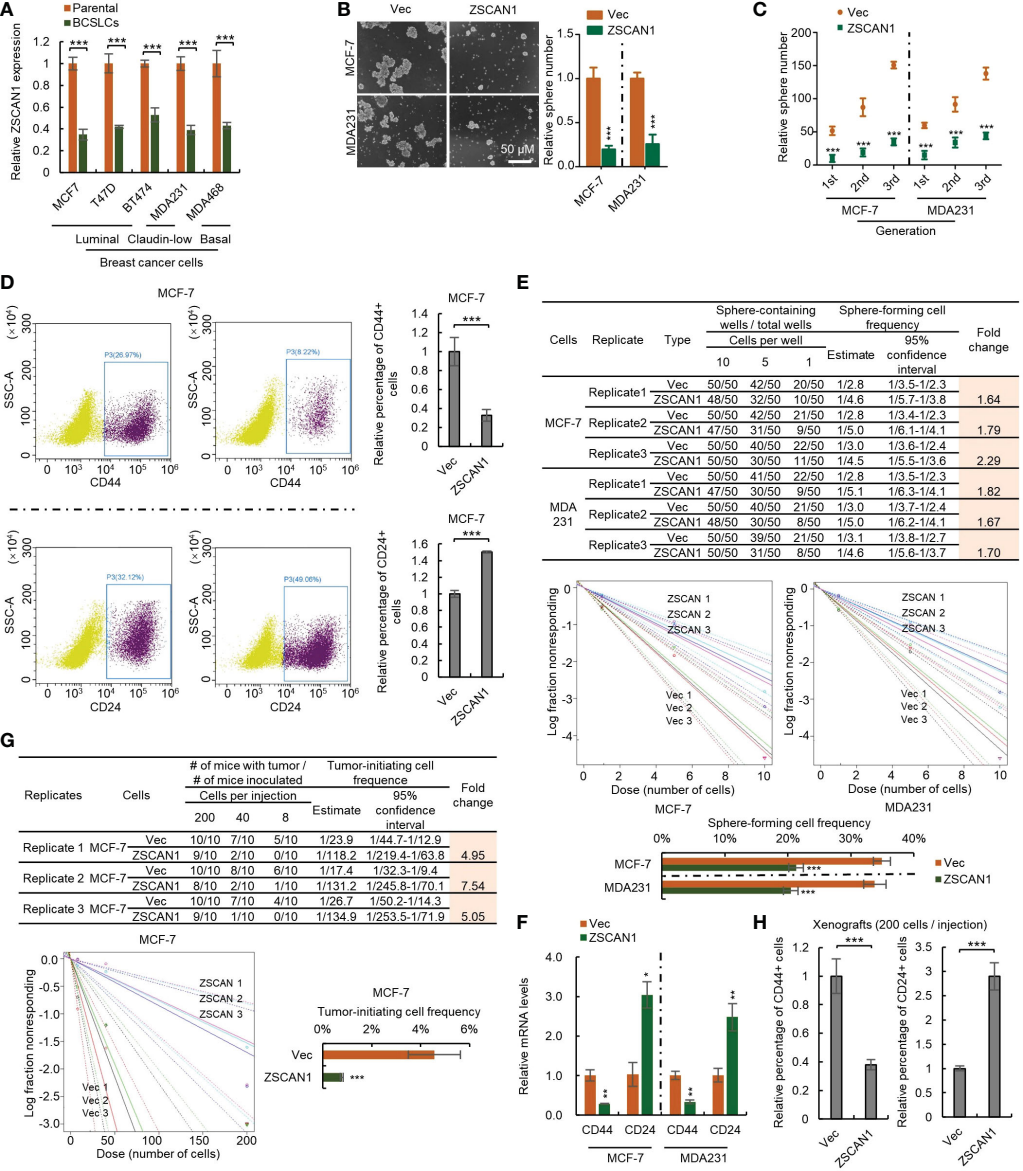
Overexpression of ZSCAN1 inhibits the stemness of BCSCs. **(A)** qRT-PCR analysis of the mRNA levels of ZSCAN1 in indicated cells (student’s *t* test, n = 3). **(B)** Overexpression of ZSCAN1 inhibited sphere-forming capacity of BCSCs as identified by sphere formation assay (student’s *t* test, n = 3). **(C)** Overexpression of ZSCAN1 inhibited serial sphere-forming capacity of BCSCs as identified by sphere formation assay (student’s *t* test, n = 3). **(D)** The percentages of CD44+ and CD24+ cells in ZSCAN1-overexpressing and control BCSCs were analyzed by flowcytometry (student’s *t* test, n = 3). **(E)** Overexpression of ZSCAN1 inhibited the frequency of sphere-forming cells of BCSCs as identified by *in vitro* limiting dilution assay (student’s *t* test). **(F)** Overexpression of ZSCAN1 downregulated the mRNA level of CD44 and upregulates the mRNA level of CD24 in BCSCs as identified by qRT-PCR (Student’s *t* test, n = 3). **(G)** Overexpression of ZSCAN1 inhibited the frequency of tumor-initiating cells of BCSCs as identified by *in vivo* limiting dilution assay (student’s *t* test). **(H)** The percentages of CD44+ and CD24+ cells in ZSCAN1-overexpressing and control xenografts were analyzed by flow cytometry (student’s *t* test, n = 3). *P < 0.05, ** *P* < 0.01, *** *P* < 0.001.

We next investigated whether ZSCAN1 is downregulated in BCSLCs. As the relatively low baseline of ZSCAN1 expression level in BC cancer cells, only qRT-PCR was adopted and the result showed that ZSCAN1 was significantly inhibited in BCSLCs versus adherent parental cells (**Figure 2A**, n =3, *P* < 0.001 in all cells), indicating the relationship between ZSCAN1 and the stemness of BCSLCs. We next examined whether BCSLCs with ZSCAN1 transfection have lower stemness properties than BCSLCs derived from empty vector control BC cells. As expected, BCSLCs derived from ZSCAN1-overexpressing BC cells formed fewer number of spheres versus BCSLCs derived from control cells (**Figure 2B**, n = 3, *P* < 0.001 in MCF-7 and MDA231). ZSCAN1-overexpressing BCSLCs also exhibited weakened serial sphere-forming capacity compared with control BCSLCs (**Figure 2C**, n = 3, *P* < 0.001 for all generations of MCF-7 and MDA231 cells). Additionally, the percentage of CD44^+^ cells of ZSCAN1-overexpressing BCSLCs was significantly decreased versus control BCSLCs (**Figure 2D**, n = 3, *P* < 0.001 in MCF-7). Oppositely, the percentage of CD24^+^ cells of ZSCAN1-overexpressing BCSLCs was significantly increased versus control BCSLCs (**Figure 2D**, n = 3, *P* < 0.001 in MCF-7). Moreover, the result from *in vitro* limiting dilution assay showed that the sphere-forming capacity was significantly decreased in ZSCAN1-overexpressing BCSLCs versus control BCSLCs (**Figure 2E**, 3 replicates, *P* < 0.001 in MCF-7 and MDA231). Furthermore, ZSCAN1-overexpressing BCSLCs exhibited decreased mRNA level of CD44 (**Figure 2F**, n =3, *P* < 0.01 in MCF-7 and MDA231) and an increased mRNA level of CD24 (**Figure 2F**, n = 3, *P* < 0.05 in MCF-7 and *P* < 0.01 in MDA231) compared with control BCSLCs. The tumor-initiating capacity of ZSCAN1-overexpressing BCSLCs was also significantly decreased versus control BCSLCs (**Figure 2G**, 3 replicates, *P* < 0.001 in MCF-7), as identified by an *in vivo* limiting dilution assay. Similarly, the percentage of CD44+ cells was significantly decreased in ZSCAN1-overexpressing xenograft (**Figure 2H**, n = 10) and the percentage of CD24+ cells was significantly increased in ZSCAN1-overexpressing xenograft (**Figure 2H**, n = 10). These results support that ZSCAN1 inhibits the stemness of BCSCs.

**Figure 3:**
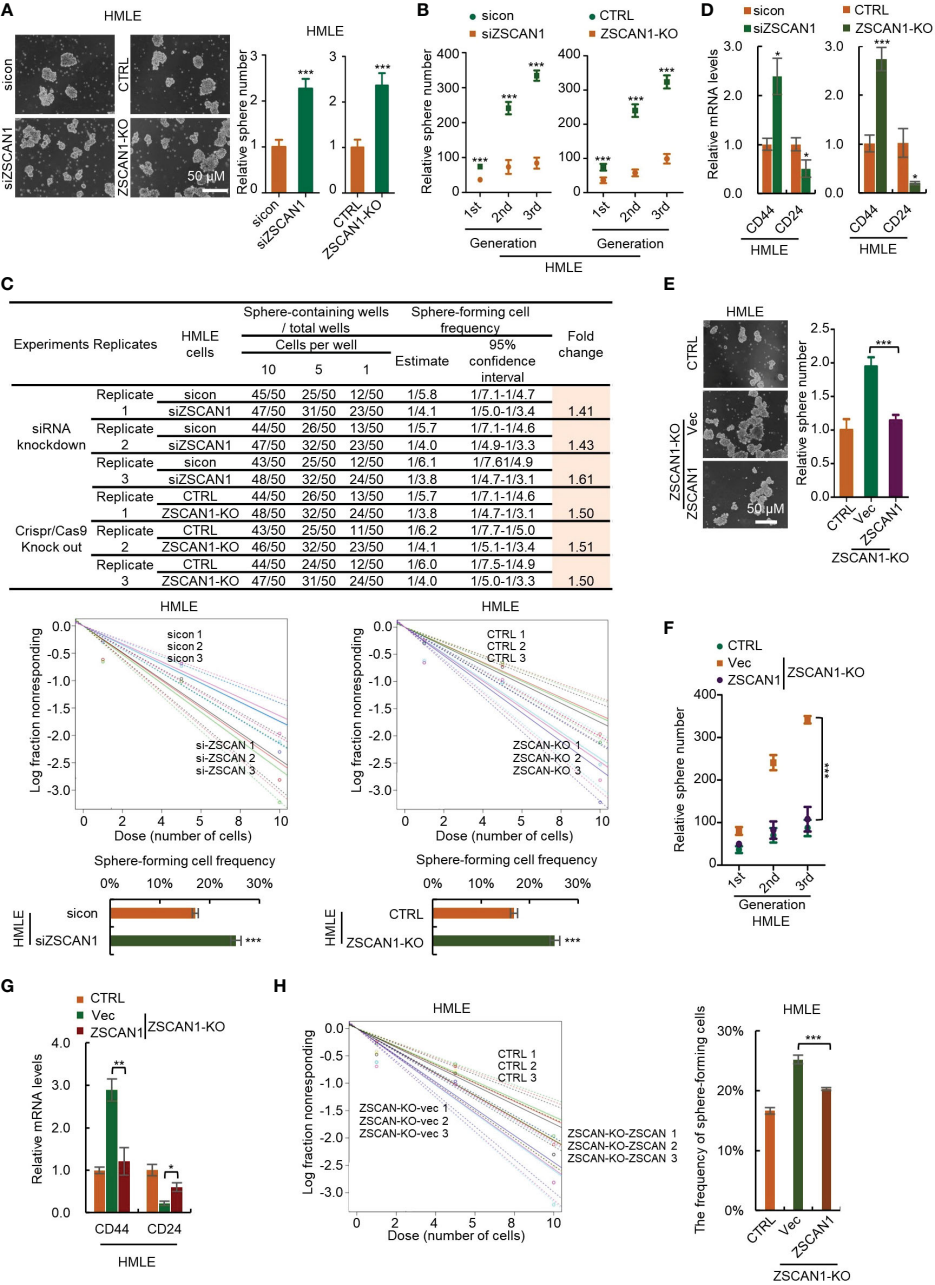
Depletion of ZSCAN1 promotes the stemness of normal human mammary epithelial cells. **(A)** Depletion of ZSCAN1 increased sphere-forming capacity of HMLE cells as identified by sphere formation assay (student’s *t* test, n = 3). **(B)** Depletion of ZSCAN1 increased serial sphere-forming capacity of HLME cells as identified by sphere formation assay (student’s *t* test, n = 3). **(C)** Depletion of ZSCAN1 increased the frequency of sphere-forming cells of HMLE cells as identified by *in vitro* limiting dilution assay (student’s *t* test). **(D)** Depletion of ZSCAN1 upregulated the mRNA level of CD44 and downregulated the mRNA level of CD24 in HMLE cells as identified by qRT-PCR (student’s *t* test, n = 3). **(E-H)** Overexpression of ZSCAN1 abolished the effect of ZSCAN1 depletion on the sphere-forming capacity **(E, F)**, the frequency of sphere-forming cells **(G)**, and the expression of BCSCs markers **(H)** in HMLE cells (one-way ANOVA, n = 3). *P < 0.05, ** *P* < 0.01, *** *P* < 0.001.

To confirm this, we depleted ZSCAN1 in normal HMLE cells (**Supplementary Figure 4A**). We found that ZSCAN1 depletion significantly increased the stemness properties of HMLEs, including sphere-forming capacity (**Figure 3A**, n = 3, *P* < 0.001 in both knockdown and knockout cells), serial sphere-forming capacity (**Figure 3B**, n = 3, *P* < 0.001 for all generations in both knockdown and knockout cells), sphere-forming frequency (**Figure 3C**, 3 replicates, *P* < 0.001 in both knockdown and knockout cells), as well as marker expression (**Figure 3D**, n =3, *P* < 0.05 and 0.001 for CD44 in knockdown and knockout cells, *P* < 0.05 for CD24 in both). Further, overexpression of ZSCAN1 in ZSCAN1-knockout cells (**Supplementary Figure 4B**) abolished the stemness inhibitory effect of ZSCAN1 depletion (**Figures 3E–H**; **Supplementary Figure 4C**), indicating that the off-target effect of ZSCAN1 depletion could be excluded. Taken together, the above results demonstrated that ZSCAN1 suppresses the stemness of BCSCs.

### ZSCAN1 inhibits the transcriptional activity of TAZ

**Figure 4:**
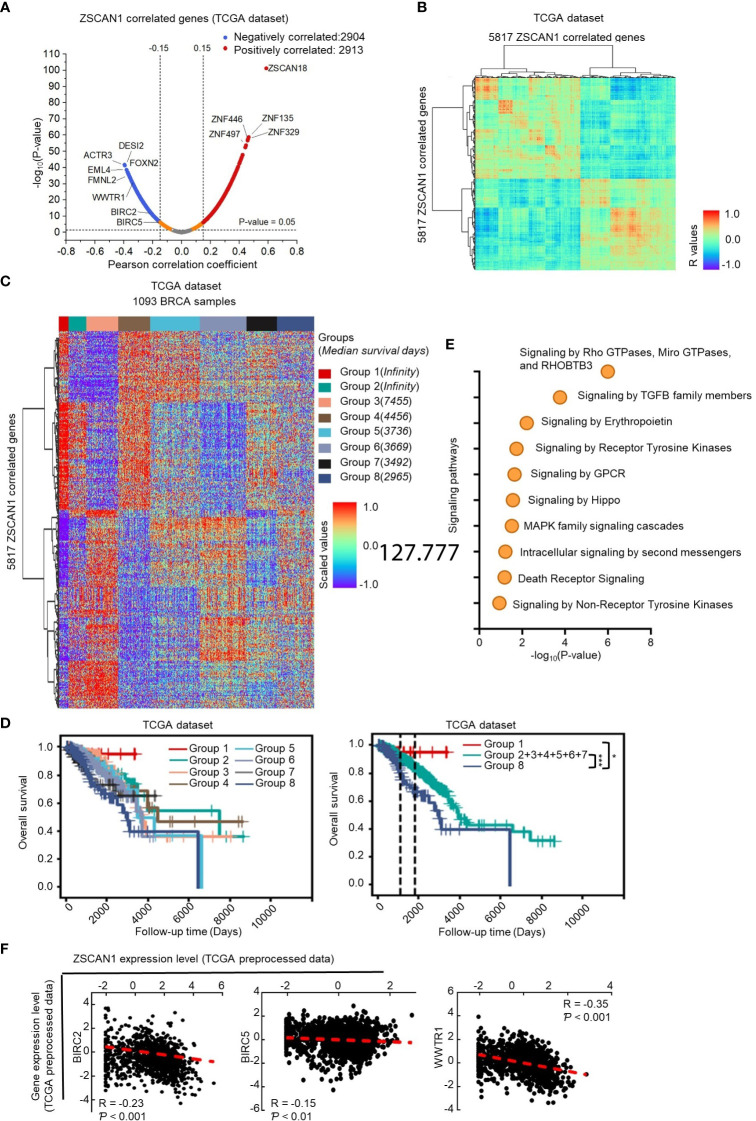
Identification of downstream targets of ZSCAN1 in breast cancer **(A)** Identification of ZSCAN1-correlated genes by Pearson analysis with TCGA dataset. **(B)** The correlation between ZSCAN1-correlated genes. **(C)** The expression profile of ZSCAN1-correlated genes in breast cancer tissues included in TCGA database. **(D)** Kaplan-meier analysis of the patients grouped by ZSCAN1-correlated genes. **(E)** Pathway analysis of genes negatively correlated with ZSCAN1. **(F)** The correlation between BIRC2, BIRC5, WWTR1, and ZSCAN1 in TCGA dataset was analyzed by Pearson analysis. *P < 0.05, *** *P* < 0.001.

Since the association of ZSCAN1 with survival outcome could be observed in BC samples included in the TCGA dataset, it is possible to screen its downstream targets by analyzing its correlated genes in the TCGA dataset. Therefore, by Pearson correlation analysis, we identified 2913 and 2904 genes positively and negatively correlate with ZSCAN1 in BC tissues by Pearson analysis (R>0.15 or<-0.15 and *P* < 0.05) (**Figure 4A**; **Supplementary Table 4**). The identified ZSCAN1 correlated genes correlate with each other (**Figure 4B**), which indicates that the ZSCAN1-correlated genes were successfully identified. The expression profile of these correlated genes in BC tissues was presented as a heatmap (**Figure 4C**); the patients were clustered into eight groups by the expression profile of ZSCAN1-correlated genes and these grouped patients exhibit distinct median survival days (**Figure 4C**). Kaplan-Meier analysis showed that patients in Group 1 and Group 8 exhibit highest and lowest survival ability, respectively (**Figure 4D**). These data support that ZSCAN1 and its correlated genes are associated with BC patient survival.

Next, we subjected ZSCAN1 correlated genes to the Reactome online tool for pathway analysis. The top pathways impacted by all correlated genes (**Supplementary Figure 5A** top), positively correlated genes (**Supplementary Figure 5A** bottom), and negatively correlated genes (**Figure 4E**) were listed. We noticed that, in Hippo/YAP signaling pathway, the downstream target genes (BIRC2 and BIRC5) and positive regulator, TAZ (encoded by WWTR1), were negatively correlated with ZSCAN1 (**Figures 4A–F**, *P* < 0.01 for BIRC5, *P* < 0.001 for BIRC2 and WWTR1). Further, the expression of BIRC2 and BIRC5 was significantly downregulated in ZSCAN1-high BC tissues versus ZSCAN1-low BC tissues (**Supplementary Figure 5B**, *P* < 0.05 for Both). Given the important role of Hippo/YAP signaling in stemness maintenance (38), we speculated that WWTR1-mediated inhibition of Hippo/YAP signaling is important for ZSCAN1 regulating the stemness of BCSCs. We next found that, in local BC samples, the expression of BIRC2, BIRC5, and WWTR1 was negatively correlated with ZSCAN1, as identified by both immunohistochemistry (**Supplementary Figure 5C**, *P* < 0.001 for all) and qRT-PCR (**Supplementary Figure 5D**, *P* < 0.001 for all) results. Additionally, the increased mRNA levels of BIRC2, BIRC5, and WWTR1 were observed in BC cell lines versus normal cell lines (**Supplementary Figure 5E**, n =3, *P* < 0.001 for all). Moreover, downregulation of BIRC2, BIRC5, and WWTR1 was observed in ZSCAN1-overexpressing cells and xenograft versus control cells and xenograft (**Supplementary Figures 5F, G**, n = 3 for cells, n = 6 for xenograft, *P* < 0.001 for all). These results motivated us to investigate the inhibitory effect of ZSCAN1 on Hippo/YAP signaling.

**Figure 5:**
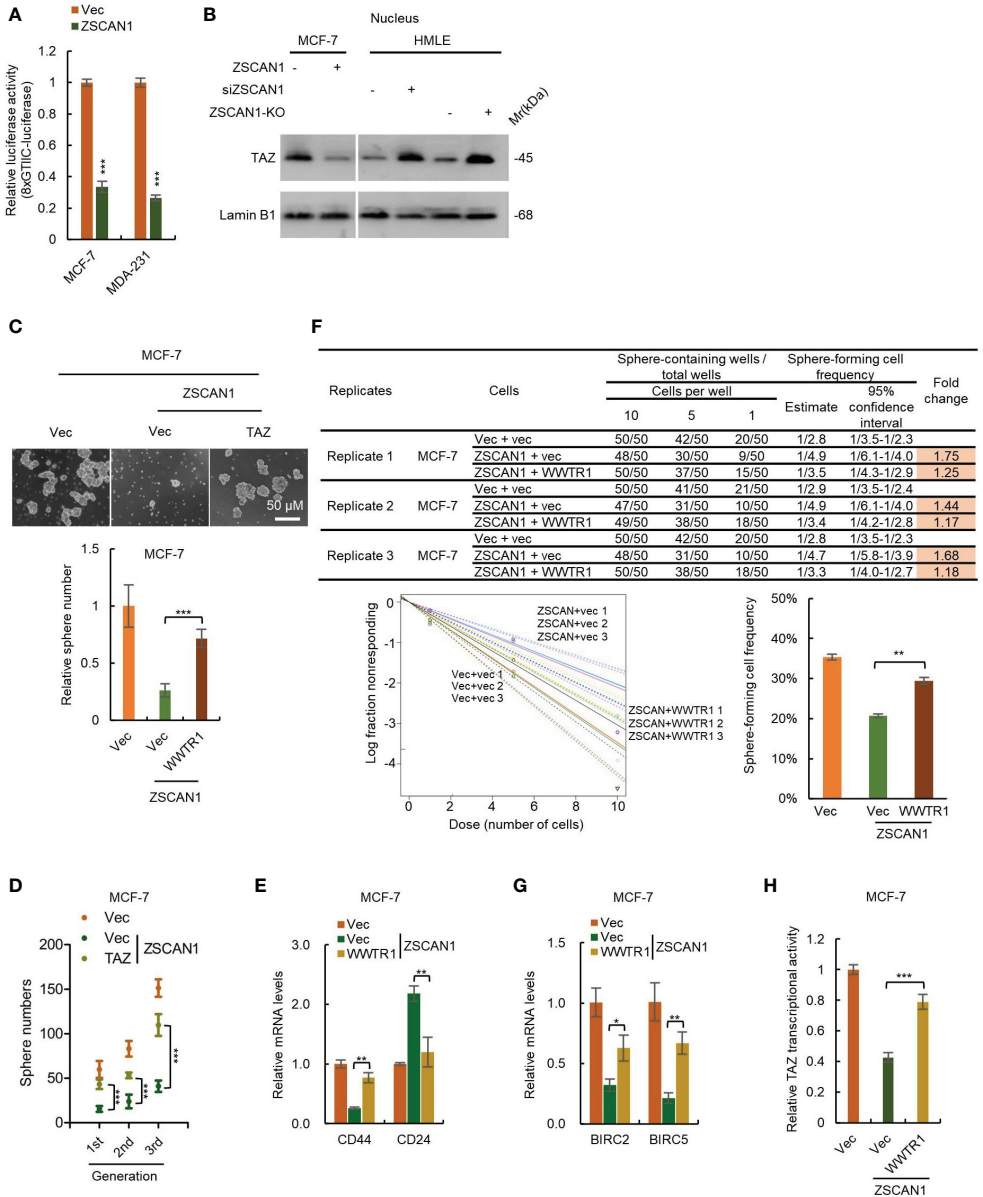
ZSCAN1 suppresses the stemness of BCSCs by inhibiting TAZ activity. **(A)** ZSCAN1 inhibited the transcriptional activity of TAZ revealed by luciferase reporter assay with an 8XGTIIC luciferase reporter plasmid containing eight TEAD consensus binding sequences (student’s *t-test*, n = 3). **(B)** ZSCAN1 negatively regulated the protein levels of TAZ in nucleus of breast cancer cells as analyzed by western blot. **(C, D)** Overexpression of TAZ (encoded by WWTR1) restored the decreased sphere-forming capacity **(C)**, serial sphere-forming capacity **(D)** in ZSCAN1-overexpressing BCSCs (one-way ANOVA, n = 3). **(E)** Overexpression of TAZ abolished the effect of ZSCAN1 on CD44 and CD24 expression in BCSCs (one-way ANOVA, n = 3). **(F)** Overexpression of TAZ restored the decreased frequency of sphere-forming cells in ZSCAN1-overexpressing BCSCs (one-way ANOVA, n = 3). **(G, H)** Overexpression of TAZ restored the decreased BIRC2 and BIRC5 expression **(G)** as well as TAZ transcriptional activity **(H)** in ZSCAN1-overexpressing BCSCs (one-way ANOVA, n = 3). *P < 0.05, ** *P* < 0.01, *** *P* < 0.001.

In mammalian cells, TAZ and YAP are two paralogous restricted by Hippo signaling; when Hippo signaling is inhibited, TAZ and YAP serve as transcriptional coactivators by binding to TEAD family of transcription factors and activate the transcription of downstream genes to enhance proliferation and stemness maintenance (38). We next employed an 8×GTIIC luciferase reporter plasmid to test the transcriptional activity of TAZ. As expected, the transcriptional activity of TAZ was significantly decreased in ZSCAN1-overexpressing cells versus control cells (**Figure 5A**, n =3, *P* < 0.001 for both). Moreover, western blot analysis showed that the abundance of nuclear TAZ was significantly decreased in ZSCAN1-overexpressing MCF-7 cells (**Figure 5B**). Together with that the mRNA levels of BIRC2 and BIRC5; were significantly decreased in ZSCAN1-overexpressing MCF-7 cells (**Supplementary Figures 5F, G**, n =3, *P* < 0.001 for all). These results support that ZSCAN1 inhibits the transcriptional activity of TAZ.

### TAZ suppression is critical for ZSCAN1 inhibiting the stemness of BCSCs

To confirm the role of TAZ suppression in ZSCAN1 inhibiting the stemness of BCSCs. We performed a rescue experiment by overexpressing TAZ in ZSCAN1-overexpressing MCF-7 cells (**Supplementary Figure 6A**). As expected, overexpression of TAZ significantly abolished the inhibitory effect of ZSCAN1 on the stemness properties of MCF-7 BCSLCs, reflected by sphere number (**Figure 5C**, n = 3, *P* < 0.001), serial sphere-forming capacity (**Figure 5D**, n = 3, *P* < 0.001), the expression of BCSCs markers (**Figure 5E**, n =3, *P* < 0.01 for CD44 and CD24), the frequency of sphere-forming cells (**Figure 5F**, 3 replicates, *P* < 0.01), the expression of BIRC2 and BIRC5 (**Figure 5G**, n =3, *P* < 0.05 for BIRC2 and *P* < 0.01 for BIRC5), as well as TAZ transcriptional activity (**Figure 5H**, n =3, *P* < 0.05). These results demonstrated that ZSCAN1 inhibits the stemness of BCSCs, at least partially, by suppressing TAZ.

### ZSCAN1 directly binds to WWTR1 promoter and inhibits TAZ at the transcriptional level

**Figure 6:**
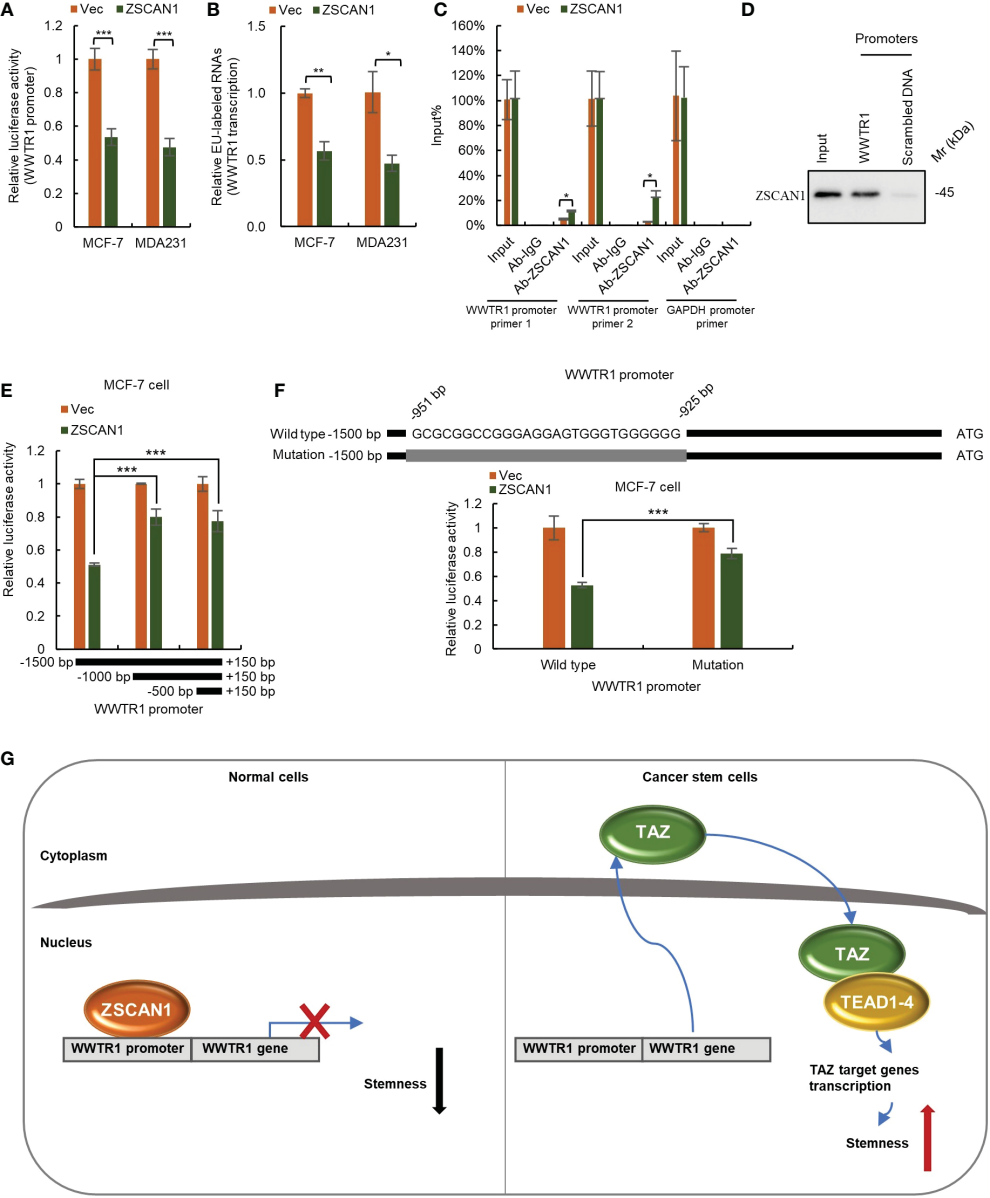
ZSCAN1 inhibits TAZ at the transcriptional level. **(A)** ZSCAN1 negatively regulated the transcriptional activity of WWTR1 (encoding for TAZ) promoters in breast cancer cells as identified by luciferase reporter assay (student’s *t-test*, n = 3). **(B)** ZSCAN1 negatively regulated the transcription of WWTR1 in breast cancer cells as identified by nuclear run-on assay (student’s *t-test*, n = 3). **(C, D)** ZSCAN1 directly bound to the promoter of WWTR1 as identified by ChIP-qPCR **(C)** (student’s *t-test*, n = 3) and DNA pull-down **(D)** assay. **(E)** The region of -1500 - -1000bp in WWTR1 promoter mediated the effect of ZSCAN1, revealed by luciferase reporter assay (student’s *t-test*, n = 3). **(F)** Depletion of -951 - -925bp region in WWTR1 promoter abolished the effect of ZSCAN1, revealed by luciferase reporter assay (student’s *t-test*, n = 3). **(G)** A schematic diagram showing ZSCAN1 suppresses the stemness of breast cancer cells by transcriptionally suppressing TAZ and TEAD4. ZSCAN1 transcriptionally inhibits TAZ and attenuates the transcriptional activity of the TAZ/TEAD4 complex, therefore suppressing the stemness of BCSCs. *P < 0.05, ** *P* < 0.01, *** *P* < 0.001.

As ZFPs family members mainly function as transcriptional regulators (10), we next investigated whether ZSCAN1 inhibits TAZ as a transcriptional repressor. We first employed luciferase reporter assay to determine whether ZSCAN1 inhibits the transcriptional activity of WWTR1 promoter. As expected, the transcriptional activity of WWTR1 promoter was significantly decreased in ZSCAN1-overexpressing cells (**Figure 6A**, n =3, *P* < 0.001 in MCF-7 and MDA231). Next, by nuclear run-on assay, we found that the nascent mRNA level of WWTR1 was significantly decreased in ZSCAN1-overexpressing cells versus control cells (**Figure 6B**, n =3, *P* < 0.001 in MCF-7 and MDA231), which confirmed that ZSCNA1 inhibits WWTR1 transcription. Next, by ChIP-PCR assay, we found that the promoter of WWTR1 could be immunoprecipitated by ZSCAN1 antibody and the amount of immunoprecipitated WWTR1 promoter was increased in ZSCAN1-overexpressing cells (**Figure 6C**, n =3, *P* < 0.001 in MCF-7 and MDA231), while the promoter of GAPDH could not be immunoprecipitated by ZSCAN1 antibody (**Figure 6C**). This result demonstrated the direct binding between ZSCAN1 and WWTR1 promoter. Additionally, the direct binding between ZSCAN1 and WWTR1 promoter was confirmed by DNA pull-down assay (**Figure 6D**). Next, by luciferase reporter assay, we found that the region of -1500 - -1000bp in WWTR1 promoter mediates the transcription inhibitory effect of ZSCAN1 (**Figure 6E**). Moreover, by searching G/C-rich sequences, the potential targets of C2H2-type zinc finger transcription factors, we found the region of -951 - -925bp in WWTR1 promoter is the potential target of ZSCAN1 (**Figure 6F**), which was subsequentially confirmed by luciferase reporter assay (**Figure 6F**, n =3, *P* < 0.001). Taken together, the above results demonstrated that ZSCAN1 directly binds to WWTR1 promoter and inhibits TAZ at the transcriptional level. Collectively, the above results demonstrated that ZSCAN1 is a novel tumor suppressor in BC, that inhibits the stemness of BCSCs by transcriptional inhibition of TAZ (**Figure 6G**).

## Discussion

In this investigation, we identified ZSCAN1 as a new tumor suppressor and transcriptional repressor that blocks TAZ’s ability to promote BC stemness. The finding of this study improved our understanding of stemness regulation in BCSCs and thus suggests a therapeutic strategy for BC caused by ZSCAN1 suppression.

Tumor tissue is composed of a heterogeneous population of cancer cells, containing at least differentiated, supportive, and tumor-infiltrating cells (39). Two models have been developed, so far, for the explanation of this intra-tumor heterogeneity, clonal evolution, and the CSC model. The former model explains that natural selection plays an essential role in the formation of sub-clonal architecture during tumor development (40). The CSCs model suggests that a small number of CSCs that possess self-renewal ability account for tumor initiation and progression, and importantly, differentiated cancer cells can be reversibly dedifferentiated under certain conditions (41). Both models emphasize the concept of cell plasticity so that stem-like cells with self-renewal capacity have been recognized as the prime therapeutic targets. Recent studies have explored several main signaling pathways that maintain the stemness of CSCs (42). However, our understanding of the mechanisms underlying CSCs maintenance is still not enough. In this study, we found that ZSCAN1 is a novel tumor suppressor, which is suppressed in breast cancer cells and predicts a better prognosis of breast cancer patients (**Figure 1**); its suppression increased the tumorigenicity and stemness properties of breast cancer cells (**Figures 1**–**3**). Furthermore, several studies have revealed the aberrant regulation in methylation and expression of ZSCAN1 genes (19–21). These results suggest that aberrant regulation of ZSCAN1 may be a result of cellular response to the environment for the acquisition of stem-like capacities.

ZNFs are composed of abundant groups of proteins that are involved in multiple essential cellular processes by different molecular mechanisms, including transcriptional regulation, DNA repair, cell migration, ubiquitin-mediated protein degradation, etc (10, 11). ZNFs plays key roles in both normal and cancer stem cells (10, 11). Furthermore, a molecular tool base on the structure of ZFPs, zinc-finger nucleases (ZFNs), has been developed for high-precision genome editing (10, 11). However, although massive studies, the roles of ZFPs were incompletely understood. Most of ZFPs serve as transcription repressors in mammalian cells (10–15). In this study, we found that ZSCAN1, which belongs to ZSCAN sub-family of ZFPS, also serves as a transcription repressor that transcriptionally inhibits WWTR1, and thus suppresses the activity of TAZ/TEAD4 complex (**Figures 5**, **6**). The study thus revealed a novel mechanism associated with stemness maintenance and ZFPs.

Hippo signaling pathway controls organ size by regulating the cellular processes associated with cell proliferation and homogeneity (43). Meanwhile, Hippo/YAP signaling is frequently used by CSCs for stemness maintenance. In Hippo signaling, unphosphorylated YAP and its paralogous, TAZ, translocated into the nucleus and form a complex with transcription factors, TEAD1-4, to regulate transcription of the target genes (38, 43). Most studies focus on the regulation of the phosphorylation and transcription of YAP, while, the regulation of TAZ was rarely studied. Here, we showed that WWTR1 is significantly inhibited by ZSCAN1 which plays an important role in the stemness homogeneity of BCSCs (**Figures 4**, **6**), and thus suggests the importance of regulation of other members in Hippo signaling. Further, Hippo/YAP signaling also plays important role in the regulation of cell proliferation (43). By rescue experiment, we found the proliferation inhibitory effect of ZSCAN1 is, at least partially, abolished by WWTR1-overexpression (**Supplementary Figure 6B**). We thus also explored the mechanism underlying ZSCAN1 suppressing the proliferation of breast cancer cells.

In summary, our findings revealed an important mechanism for BCSC maintenance: ZSCAN1, a novel tumor suppressor and transcriptional repressor, inhibits BCSC stemness *via* TAZ suppression. Our finding thus improved our understanding of stemness regulation in BCSCs and suggests a therapeutic strategy for breast cancer caused by ZSCAN1 suppression.

## Data availability statement

The raw data supporting the conclusions of this article will be made available by the authors, without undue reservation.

## Ethics statement

The studies involving human participants were reviewed and approved by Institutional Review Board of the Second Affiliated Hospital of Soochow University. The patients/participants provided their written informed consent to participate in this study. The animal study was reviewed and approved by Institutional Animal Care and Use Committee in The Second Affiliated Hospital of Soochow University.

## Author contributions

TiL and GJ designed the research. JC, YL, MH and HZ performed most of the experiments. JC, HZ, YL, JL, and CC performed the experiments involving cell- and animal-based experiments. MY performed bioinformatic analysis. JC, LH, XH, TaL, CL, and HF performed the experiments associated with clinical specimens. LZ performed data analysis and bioinformatic study. TiL and GJ supervised the study and wrote the manuscript. All authors contributed to the article and approved the submitted version.
