# Supplementary material for: Zinc finger and SCAN domain containing 1, ZSCAN1, is a novel stemness-related tumor suppressor and transcriptional repressor in breast cancer targeting TAZ
Source: Front Oncol. 2023 Feb 27;13:1041688. doi: 10.3389/fonc.2023.1041688 (PMC10009259; doi:10.3389/fonc.2023.1041688)
Supplement: Supplementary file 1 [file DataSheet_1.pdf]

Supporting information

Zinc Finger and SCAN Domain Containing 1, ZSCAN1, is a novel stemness-related tumor suppressor and transcriptional repressor in breast cancer targeting TAZ

Jian Chu[1†], Yunzhe Li[2†], Misi He[3], Hui Zhang[4], Lingling Yang[5], Muyao Yang[2], Jingshu Liu[6], Chenxi Cui[5], Liquan Hong[7], Xingchi Hu[8], Lei Zhou[9,10], Tangya Li[8], Changchun Li[8], Huiwen Fan[8], Guoqin Jiang[1*], Tingyuan Lang[3,11*]

[1] Department of Surgery, The Second Affiliated Hospital of Soochow University, Suzhou, Jiangsu 215004, People's Republic of China.

[2] College of Bioengineering, Chongqing University, Chongqing 400044, People's Republic of China.

[3] Department of Gynecologic Oncology, Chongqing University Cancer Hospital & Chongqing Cancer Institute & Chongqing Cancer Hospital, Chongqing 400030, Chongqing, People's Republic of China.

[4] Department of Breast Cancer Center, Chongqing University Cancer Hospital, Chongqing 400030, People's Republic of China.

[5] School of Medicine, Chongqing University, Chongqing 400044, Chongqing, People's Republic of China.

[6] Obstetrics and Gynecology Department, The Second Affiliated Hospital of Chongqing Medical University, Chongqing 400010, People's Republic of China.

[7] Department of Clinical Laboratory, Affiliated Hospital of Hangzhou Normal University, Hangzhou, Zhejiang 310015, People's Republic of China.

[8] Department of General Surgery, Yancheng City No.1 People’s Hospital, Yancheng, Jiangsu 224005, People's Republic of China.

[9] School of Optometry; Department of Applied Biology and Chemical Technology; Research Centre for SHARP Vision (RCSV); The Hong Kong Polytechnic University, Hong Kong.

[10] Centre for Eye and Vision Research (CEVR), 17W Hong Kong Science Park, Hong Kong.

[11] Reproductive Medicine Center, The First Affiliated Hospital of Chongqing Medical University, Chongqing 400042, Chongqing, People's Republic of China.

[†]Jian Chu and Yunzhe Li contributed equally to this work

***Correspondence:** Tingyuan Lang (michaellang2009@163.com) and Guoqin Jiang (jiang_guoqin@163.com)

Supplementary Table S1. Antibody used in the study.

Antigen	Antibody	Conjugated with	Application	Manufacture	Catalog #	Dilution (1:)
ZSCAN1	Rabbit, IgG	Unconjugated	WB, IHC, ChIP	ThermoFisher	PA5-41696	1800 for WB, 800 for IHC, 500 for ChIP
TAZ	Rabbit, IgG	Unconjugated	WB	CST	#72804	1500 for WB, 500 for IHC
TEAD4	Rabbit, IgG	Unconjugated	WB	Abcam	ab151274	1200 for WB, 200 for IHC
BIRC2	Rabbit, IgG	Unconjugated	WB	CST	#70008	1000 for WB, 500 for IHC
BIRC5	Rabbit, IgG	Unconjugated	WB	CST	#2808	1000 for WB, 200 for IHC
Ki67	Rabbit, IgG	Unconjugated	WB	Abcam	Ab16667	2000 for WB, 500 for IHC
β-actin	Rabbit, IgG	Unconjugated	WB	Abcam	ab8226	3000
Rabbit IgG	Goat, NA	HRP	WB	CST	#7074	2000

CST: Cell Signaling Technology, WB: Western blot, HRP: horseradish peroxidase, IHC

Supplementary Table S2. Primers used in this study.

**Reverse transcription PCR**	
ZSCAN1 CDs	F: 5'-ATGCTTCCACGGCCCAAAGCCC-3'
	R: 5'-TCACATGTGGCCGTGGGCCGTGTGG-3'
WWTR1 CDs	F: 5'-ATGAATCCGGCCTCGGCGCCCCC-3'
	R: 5'-TTACAGCCAGGTTAGAAAGGGCT-3'
TEAD4 CDs	F: 5'-TTGGAGGGCACGGCCGGCACCA-3'
	R: 5'-TCATTCTTTCACCAGCCTGTAG-3'
TAZ promoter (-1500bp-+150bp)	F: 5'-CCCAGCTTTTTCTTCTCAATCTCTAGTG-3'
	R: 5'-CTCCGGCAGGATCTTCTTCCGC-3'
TAZ promoter (-1000bp-+150bp)	F: 5'-CGTGGTAAACTCAAAGGAATGCAGATGC-3'
	R: 5'-CTCCGGCAGGATCTTCTTCCGC-3'
TAZ promoter (-500bp-+150bp)	F: 5'-GCGGCCGCCGCGCGCTCAGGCTCAGCTT-3'
	R: 5'-CTCCGGCAGGATCTTCTTCCGC-3'
TEAD4 promoter (-1500bp-+150bp)	F: 5'-TCTCCCACGAGCTCCTGGTAACCAC-3'
	R: 5'-ACTCTGCTCAATATCCGGGCTCCA-3'
TEAD4 promoter (-1000bp-+150bp)	F: 5'-TGTGCCTGGCCTAATGTGACTGCT-3'
	R: 5'-ACTCTGCTCAATATCCGGGCTCCA-3'
TEAD4 promoter (-500bp-+150bp)	F: 5'-GGTTTCACCATGTTGGCCAGGCTGG-3'
	R: 5'-ACTCTGCTCAATATCCGGGCTCCA-3'
**Real-Time Quantitative Reverse Transcription PCR**	
ZSCAN1	F: 5'-TCCCCGAGGAAAGTGAGTG-3'
	R: 5'-CTCAGGCTCGTCCCAGATACT-3'
Ki67	F: 5'-GCCTGCTCGACCCTACAGA-3'
	R: 5'-GCTTGTCAACTGCGGTTGC-3'
BIRC2	F: 5'-GTTCAGTGGTTCTTACTCCAGC-3'
	R: 5'-ACTGTAGGGGTTAGTCCTCGAT-3'
BIRC5	F: 5'-AGGACCACCGCATCTCTACAT-3'
	R: 5'-AAGTCTGGCTCGTTCTCAGTG-3'
CD44	F: 5'-CTGCCGCTTTGCAGGTGTA-3'
	R: 5'-CATTGTGGGCAAGGTGCTATT-3'
CD24	F: 5'-CTCCTACCCACGCAGATTTATTC-3'
	R: 5'-AGAGTGAGACCACGAAGAGAC-3'
WWTR1	F: 5'-TCCCAGCCAAATCTCGTGATG-3'
	R: 5'-AGCGCATTGGGCATACTCAT-3'
TEAD4	F: 5'-GGACACTACTCTTACCGCATCC-3'
	R: 5'-TCAAAGACATAGGCAATGCACA-3'
WWTR1 promoter	F: 5'-CCAGGTAGTGGGGCGTGGAGCT-3'
	R: 5'-GGGGTGGAGGGAATAACTGCAG-3'
TEAD4 promoter	F: 5'-CTCGGGGGATGTGAGAGCCG-3'
	R: 5'-CTCCAGCGGGCACTACCTGG-3'
GAPDH	F: 5'-CTGGGCTACACTGAGCACC-3'
	R: 5'- AAGTGGTCGTTGAGGGCAATG-3'

Supplementary Table S3. gRNAs used in this study.

Name	Sequence
ZSCAN1 #1	ACTGCCGGAAGCGCAGACGC
ZSCAN1 #2	GCACCAGAAGACCCATCGCG
ZSCAN1 #3	CTAGGCGTCTGAGCTGATTC
Non-target control	CCATCACCGATCGTGAGCCT

Supplementary Figures:

Supplementary Figure S1

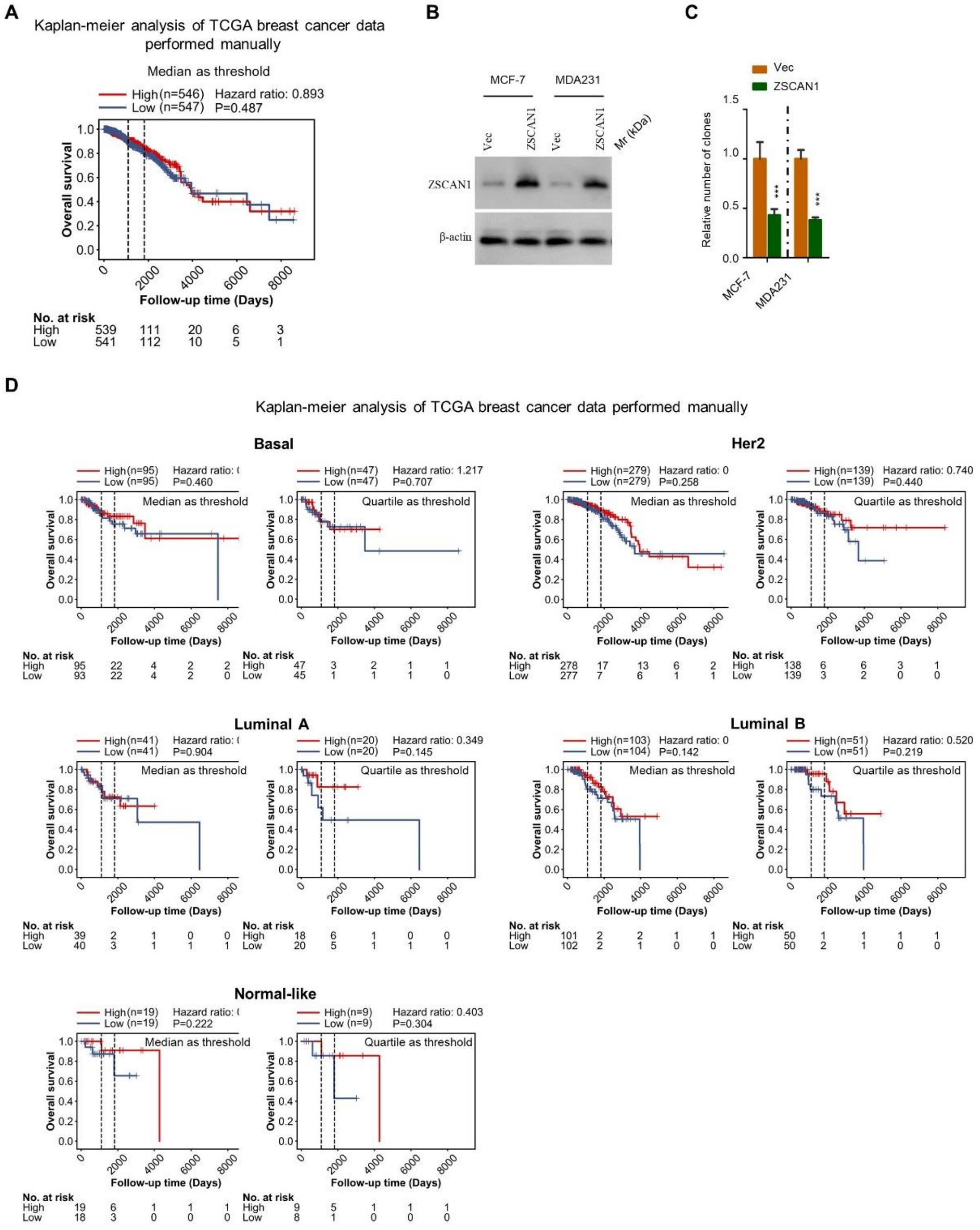

Supplementary Figure S1. Association between ZSCAN1 and the survival of breast cancer patients.

(A) Kaplan-meier analysis of the association between ZSCAN1 and the survival of all breast cancer patients included in TCGA dataset. (B) Characterization of ZSCAN1-overexpressing cells. (C) Analysis of clonogenicity of ZSCAN1-overexpressing and control cells. (D) Kaplan-meier analysis of the association between ZSCAN1 and the survival of subtypes of breast cancer patients.

Supplementary Figure S2

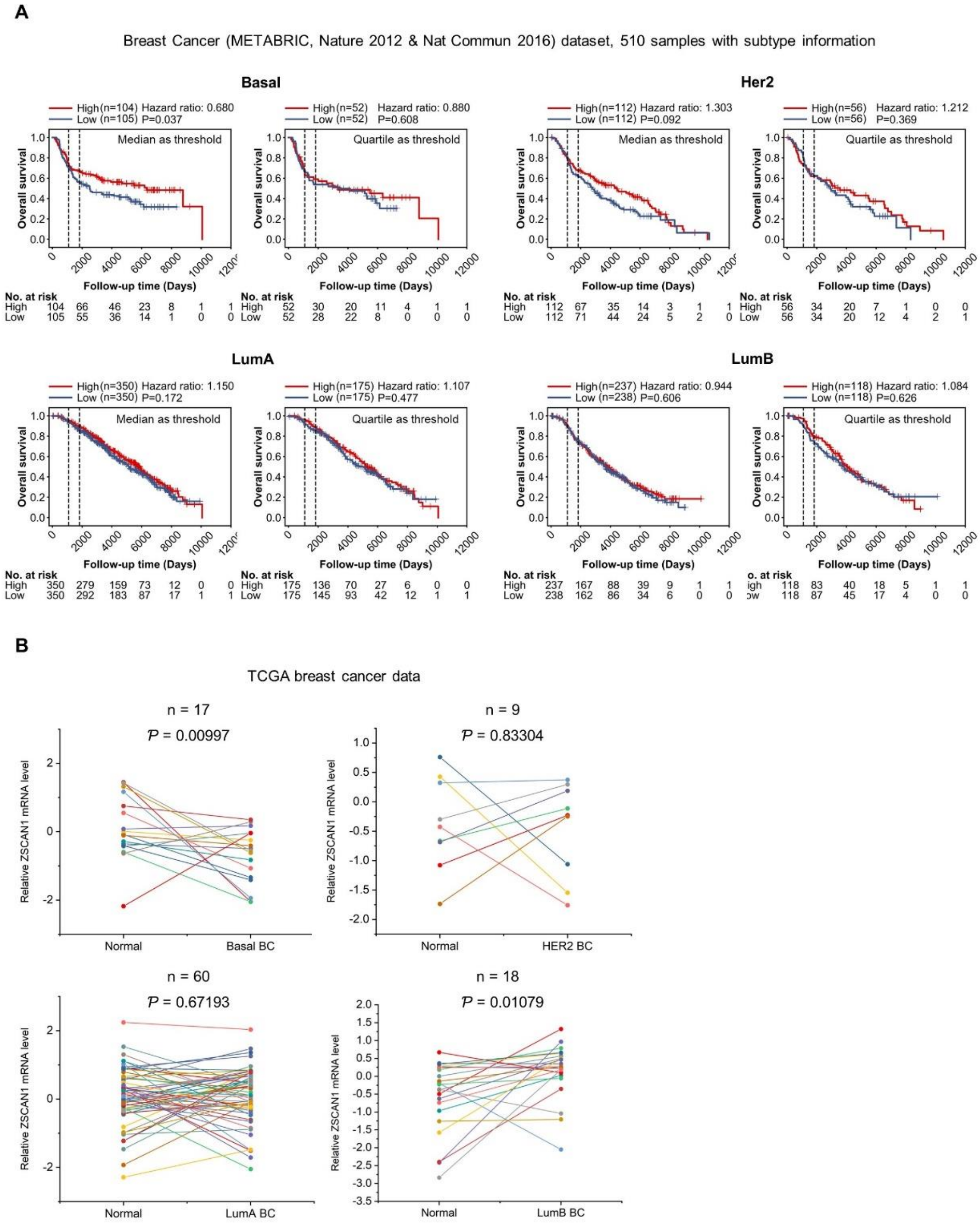

Supplementary Figure S2. Association between ZSCAN1 and the survival of different subtypes of breast cancer patients.

(A) Kaplan-meier analysis of the association between ZSCAN1 and the survival of subtypes of breast cancer patients with METABRIC dataset. (B) Expression of ZSCAN1 in different subtypes of breast cancer tissues versus normal tissues.

Supplementary Figure S3

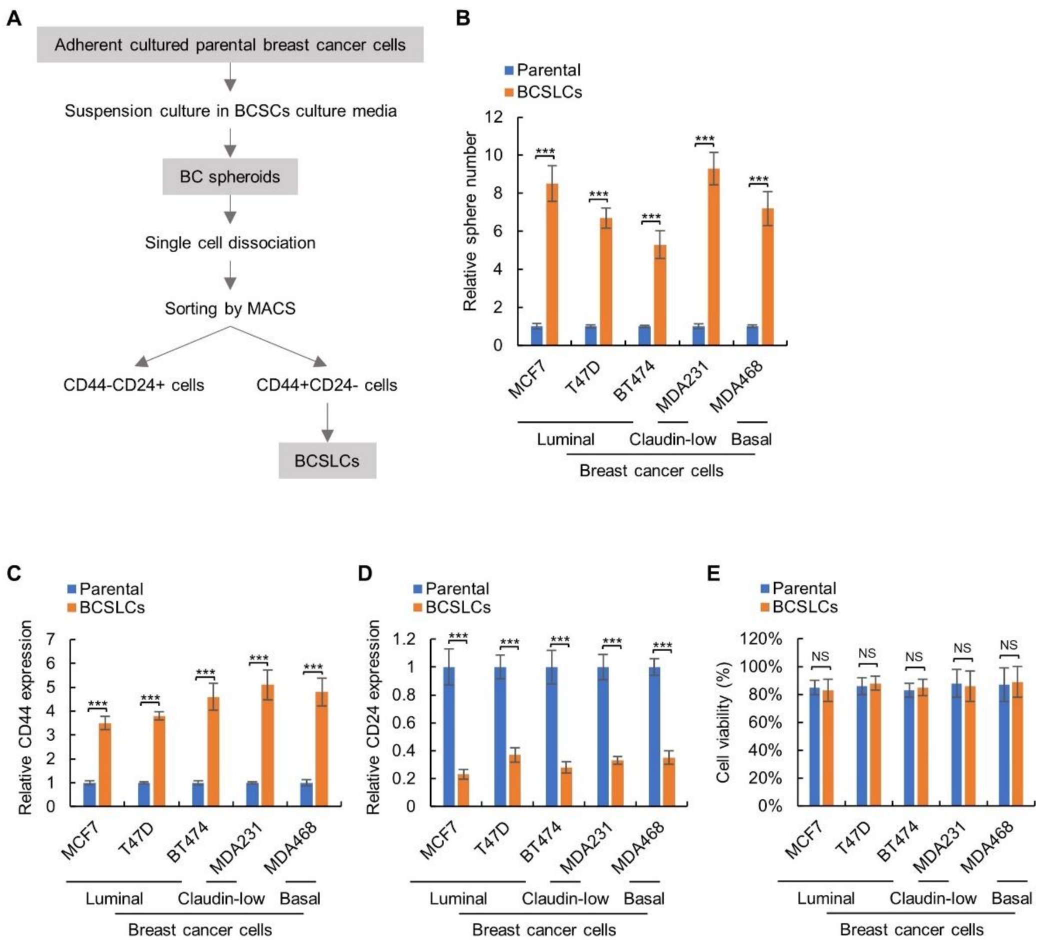

Supplementary Figure S3. Characterization of breast cancer stem-like cells.

(A) Workflow of breast cancer stem-like cells isolation. (B) Analysis of the sphere-forming capacity of breast cancer stem-like cells versus normal cells. (C,D) qRT-PCR analysis the expression of CD44 (C) and CD24 (D) in indicated cells. (E) Examination of cell viability of indicated cells.

Supplementary Figure S4

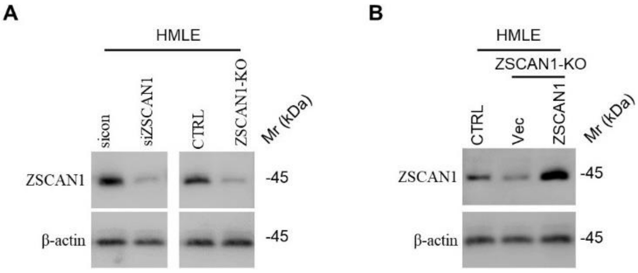

C

Replicates	HMLE cells		Sphere-containing wells / total wells: Cells per well 10	5	1	Sphere-forming cell frequency: Estimate	95% confidence interval	Fold change
Replicate 1	CTRL		45/50	25/50	12/50	1/5.8	1/7.1-1/4.7	
	ZSCAN1-KO	Vec	47/50	31/50	23/50	1/4.1	1/5.0-1/3.4	1.41
		ZSCAN1	46/50	27/50	18/50	1/5.0	1/6.1-1/4.0	1.16
Replicate 2	CTRL		44/50	25/50	11/50	1/6.0	1/7.5-1/4.9	
	ZSCAN1-KO	Vec	48/50	32/50	23/50	1/3.9	1/4.7-1/3.2	1.54
		ZSCAN1	46/50	28/50	17/50	1/4.9	1/6.0-1/4.0	1.22
Replicate 3	CTRL		43/50	24/50	12/50	1/6.2	1/7.8-1/5.0	
	ZSCAN1-KO	Vec	47/50	32/50	25/50	1/3.9	1/4.8-1/3.2	1.59
		ZSCAN1	45/50	28/50	19/50	1/4.9	1/6.1-1/4.0	1.27

Supplementary Figure S4: ZSCNA1 depletion increased the stemness properties of normal breast cells.
(A,B) Characterization of ZSCAN1-knockdown, ZSCAN1-knockout, and ZSCAN1-overexpressing ZSCAN1-knockout cells. (C) Limiting dilution assay revealed the sphere-forming frequency of indicated cells.

Supplementary Figure S5

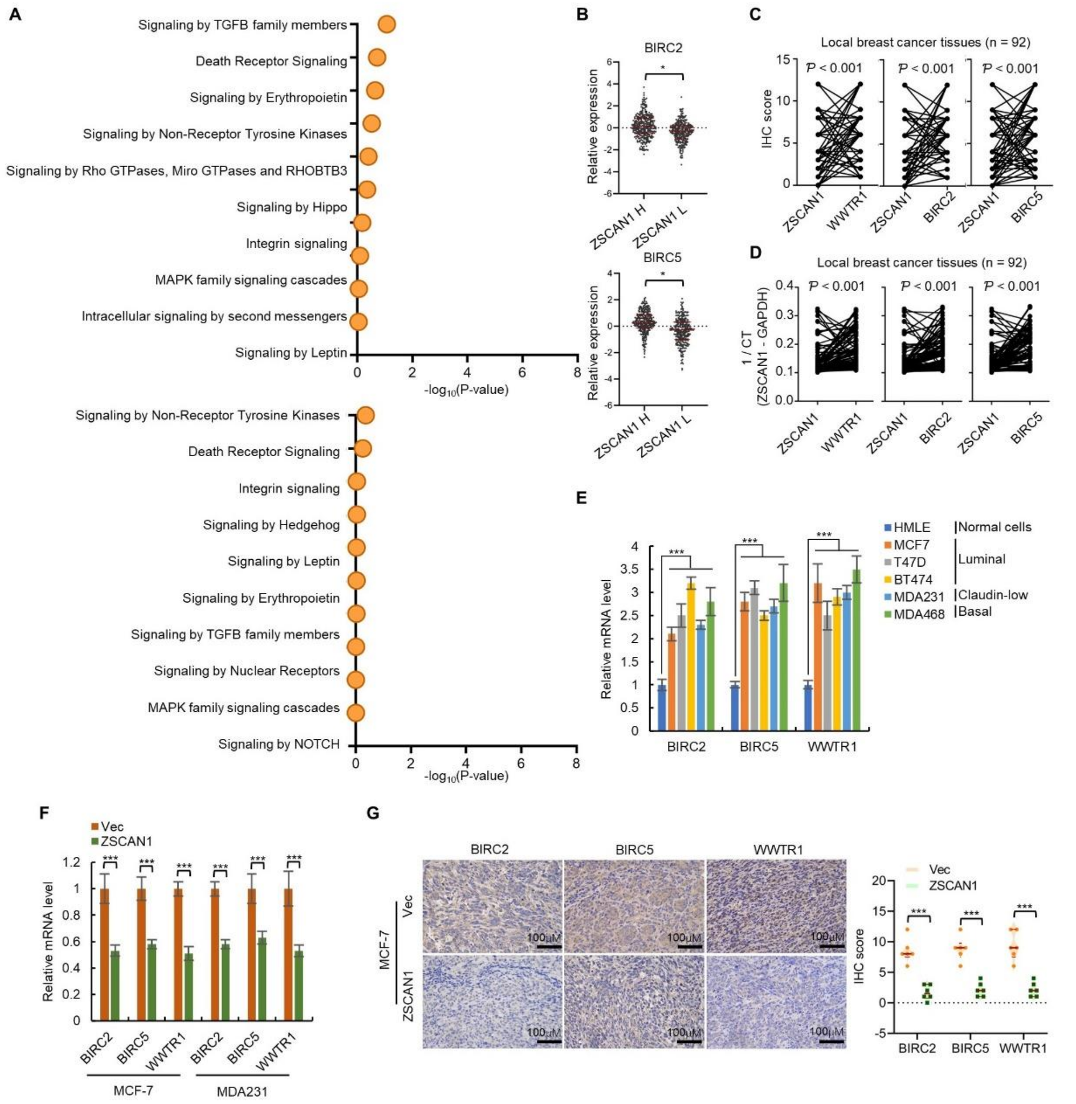

Supplementary Figure S5: Hippo/YAP signaling is inhibited by ZSCAN1. (A) Pathways impacted by ZSCAN1-correlated genes. (B) Expression of BIRC2 and BIRC5 in ZSCAN1-high and ZSCAN1-low tissues. (C,D) Correlation between BIRC2, BIRC5, WWTR1 and ZSCAN1 in local breast cancer tissues. (E) qRT-PCR analysis of BIRC2, BIRC5, and WWTR1 in normal and breast cancer cells. (F) qRT-PCR analysis of BIRC2, BIRC5, and WWTR1 in ZSCAN1-overexpressing and control cells. (G) Immunohistochemistry analysis of BIRC2, BIRC5, and WWTR1 in ZSCAN1-overexpressing and control xenografts.

Supplementary Figure S6

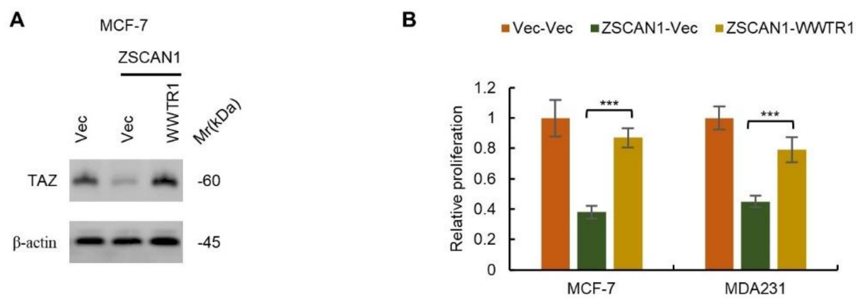

Supplementary Figure S6: ZSCAN1 regulates the proliferation of breast cancer cells by regulating WWTR1.
(A) Characterization of WWTR1-overexpressing ZSCAN1-overexpressing cells. (B) Determination of proliferation of indicated cells.
